# Engineering MXene Nanomaterials: Structure–Property Relationships, Functional Design, and Emerging Technologies

**DOI:** 10.3390/nano16150945

**Published:** 2026-07-31

**Authors:** Huy Loc Nguyen, Thi Bich Ngoc Nguyen

**Affiliations:** 1 Department of Engineering and Technology, Van Hien University, Ho Chi Minh City 72419, Vietnam; 2 Department of Water Management and Hydrological Sciences, Texas A&M University, 400 Bizzell St, College Station, TX 77843, USA; nguyent0313@tamu.edu

**Keywords:** MXenes, two-dimensional nanomaterials, structure–property relationships, surface functionalization, MXene heterostructures, emerging technologies

## Abstract

MXenes have emerged as a rapidly expanding family of two-dimensional transition-metal carbides, nitrides, and carbonitrides, characterized by exceptional compositional diversity, tunable surface chemistry, metallic conductivity, hydrophilicity, mechanical flexibility, and rich redox activity. These characteristics make MXenes highly attractive for next-generation technologies, including energy storage and conversion, catalysis, electromagnetic interference shielding, sensors, water purification, biomedical systems, and smart functional devices. However, the performance of MXene-based materials is strongly governed by their synthesis routes, defect structures, interlayer spacing, surface terminations, oxidation stability, and interfacial interactions with polymers, metals, oxides, and other two-dimensional materials. Therefore, a structure–property-oriented understanding is essential for moving MXene research from empirical material development toward rational functional design. Unlike application-centered summaries, this review develops a cross-application engineering framework that connects MXene synthesis and processing with multiscale structure, functional properties, performance trade-offs, and translational requirements. First, major synthesis and processing strategies are discussed, including selective etching, delamination, intercalation, surface modification, and scalable fabrication. Next, the relationships between MXene composition, morphology, surface chemistry, electrical conductivity, electrochemical behavior, mechanical properties, and environmental stability are analyzed. Recent advances in functionalization, heterostructure construction, and composite engineering are then highlighted to illustrate how MXene properties can be tailored for emerging applications. Finally, key challenges related to oxidation, restacking, long-term stability, environmental safety, reproducibility, and industrial translation are critically evaluated. This review aims to establish a design framework for engineering MXene nanomaterials toward high-performance, stable, and scalable emerging technologies.

## 1. Introduction

Two-dimensional (2D) nanomaterials have reshaped modern materials science by providing atomically thin platforms with large surface areas, tunable electronic structures, and strong interfacial activity [1]. Since the isolation of graphene, considerable attention has been directed toward expanding the family of 2D materials beyond carbon-based systems to include transition-metal dichalcogenides, boron nitride, layered double hydroxides, phosphorene, covalent organic frameworks, and metal–organic frameworks. Among these materials, MXenes have attracted exceptional interest because they combine several features that are rarely present in a single 2D platform, including high electrical conductivity, hydrophilicity, compositional diversity, rich surface chemistry, redox activity, mechanical flexibility, and compatibility with solution-based processing [2]. These properties have positioned MXenes as promising candidates for energy storage, electrocatalysis, electromagnetic interference shielding, sensing, water purification, biomedical systems, and smart functional devices [3,4].

MXenes are generally represented by the formula M_n+1_X_n_T_x_, where M is an early transition metal, X is carbon and/or nitrogen, and T_x_ denotes surface terminations such as –O, –OH, –F, or –Cl [5]. This structural motif gives MXenes a distinctive advantage over many other 2D materials: their properties can be engineered not only through elemental composition and layer thickness, but also through surface terminations, interlayer spacing, defect chemistry, oxidation state, and interfacial interactions. As a result, MXenes are not a single material class with fixed behavior, but a highly tunable family of nanomaterials whose performance depends strongly on synthesis, processing, functionalization, and environmental conditions. This tunability creates broad opportunities for functional design, but it also introduces significant complexity in understanding and predicting structure–property relationships [6].

Continuous advances in synthesis and processing have driven the rapid expansion of MXene research. Selective etching of layered MAX phases remains the most established route for producing MXenes. In contrast, alternative strategies such as molten salt etching, electrochemical etching, fluoride-free synthesis, bottom-up growth, intercalation-assisted delamination, and scalable dispersion processing have expanded the accessible compositions and morphologies [7]. These developments have improved control over layer number, lateral size, surface chemistry, colloidal stability, and defect density. However, they have also revealed that small differences in synthesis conditions can strongly influence conductivity, electrochemical performance, oxidation stability, mechanical behavior, and long-term reliability. Therefore, MXene synthesis should not be viewed simply as a preparative step, but as a primary engineering tool that determines material functionality from the earliest stage of design [8].

A central challenge in the MXene field is that exceptional performance in one application does not automatically translate into general material superiority. For example, high electrical conductivity may be desirable for flexible electronics, electromagnetic shielding, and current collectors, whereas controlled surface redox activity and ion accessibility may be more important for batteries, supercapacitors, and catalysis [9]. Similarly, hydrophilicity can improve aqueous dispersibility and processability, but excessive surface oxidation may compromise stability and performance during storage or operation. Restacking of MXene nanosheets can limit ion transport and reduce accessible active sites, while aggressive delamination or surface modification may introduce defects that either enhance reactivity or weaken structural integrity. These trade-offs highlight the need for a design framework that connects MXene structure, surface chemistry, processing history, and application-specific performance [10].

Surface functionalization and composite engineering have become particularly important for addressing these challenges. By modifying terminal groups, introducing heteroatoms, controlling interlayer species, constructing heterostructures, or integrating MXenes with polymers, oxides, metals, carbon materials, and other 2D nanomaterials, researchers can tailor electronic transport, mechanical strength, catalytic activity, adsorption behavior, and environmental stability [2]. Such strategies have enabled MXene-based electrodes with improved ion diffusion, catalysts with more accessible active sites, membranes with enhanced selectivity, sensors with higher sensitivity, and polymer composites with superior conductivity or shielding performance [11,12]. Nevertheless, functionalization must be carefully controlled because excessive modification can block active surfaces, disrupt conductivity, reduce processability, or introduce new safety and reproducibility concerns [13].

Despite substantial progress, several unresolved issues continue to limit the transition of MXenes from laboratory-scale demonstrations to reliable technological platforms. Oxidation and hydrolysis remain major barriers, particularly for aqueous dispersions, thin films, and devices exposed to oxygen, moisture, heat, or biological environments. Batch-to-batch variability, incomplete control over surface terminations, residual etchants or intercalants, and inconsistent reporting of synthesis conditions complicate comparison across studies [14]. In addition, many MXene-based devices are evaluated under idealized laboratory conditions, whereas real applications require long-term stability, scalable manufacturing, environmental compatibility, mechanical durability, and cost-effective processing [15]. These limitations are particularly important for emerging technologies, such as MXenes, where they are expected to function under complex electrochemical, thermal, mechanical, or biological conditions.

Several representative reviews have established important foundations for understanding MXene nanomaterials, but their objectives and organizational frameworks differ from those adopted here. Akhter et al. (2023) [16] comprehensively reviewed MXene synthesis and properties with particular emphasis on supercapacitor applications, whereas Borah et al. (2025) [17] provided a broad survey of recent developments in MXene synthesis, properties, and applications. More recently, Al-Ghubairi et al. (2026) [18] examined synthesis and surface-engineering strategies for advanced functional applications. In contrast, the present review is organized around an engineering sequence that links synthesis and processing conditions, with multiscale structural variables, including composition, surface terminations, defects, interlayer chemistry, flake morphology, and interfacial architecture, and subsequently connects these variables to functional properties and application performance. Research interests focus on competing design requirements, such as conductivity versus ion accessibility, surface reactivity versus oxidation stability, and functional enhancement versus processability and reproducibility. The specific contribution of this review is therefore a cross-application structure–property–performance framework that integrates functional design with stability, scalable manufacturing, safety, sustainability, and data-driven material development rather than presenting another application-by-application catalogue of MXene studies.

Building on this distinction, the review follows a synthesis and processing–structure and interface–property–performance framework for the engineering of MXene nanomaterials. Rather than exhaustively cataloguing reported applications, it examines how controllable variables, such as composition, synthesis route, surface termination, defect structure, interlayer environment, flake assembly, functionalization, and heterostructure construction, generate application-specific properties, benefits, and trade-offs. The review first introduces the structural characteristics and design logic of MXenes, then discusses how composition and surface chemistry influence electrical, electrochemical, mechanical, catalytic, adsorption, and stability-related properties. Recent advances in functionalization and composite engineering are then analyzed to illustrate how MXene performance can be tailored for energy, environmental, sensing, biomedical, and smart device applications. Finally, key challenges and future opportunities related to oxidation control, reproducibility, safety, sustainability, scalable manufacturing, and data-driven material design are highlighted. By emphasizing structure–property relationships and functional engineering, this review aims to provide a coherent framework for guiding the next stage of MXene research toward stable, high-performance, and technologically relevant nanomaterials.

## 2. Methodologies

This review was prepared through a structured literature search and critical analysis of peer-reviewed publications related to MXene nanomaterials, with emphasis on synthesis, surface chemistry, structure–property relationships, functionalization strategies, and emerging applications. Relevant studies were collected from major scientific databases, including Web of Science, Scopus, ScienceDirect, PubMed, SpringerLink, Wiley Online Library, ACS Publications, and Google Scholar.

The literature search used combinations of keywords such as “MXene,” “two-dimensional materials,” “MXene synthesis,” “surface termination,” “MXene functionalization,” “structure–property relationships,” “MXene composites,” “MXene heterostructures,” “energy storage,” “catalysis,” “sensing,” “water purification,” “biomedical applications,” and “emerging technologies.” Priority was given to original research articles published in recent years, particularly studies that reported clear relationships between MXene structure, processing conditions, surface chemistry, material properties, and application performance. Highly cited foundational papers and selected review articles were also included when necessary to provide background context.

Studies were analyzed according to MXene composition, synthesis route, morphology, surface termination, interlayer structure, functionalization strategy, physicochemical properties, application area, and translational relevance. Attention was given to reports that connected material design with performance outcomes, stability, scalability, reproducibility, and practical limitations. The selected literature was then organized to identify major advances, unresolved challenges, and future research directions for engineering MXene nanomaterials toward advanced technological applications.

## 3. Structural Characteristics, Synthesis Logic, and Processing Design of MXene Nanomaterials

### 3.1. Structural Identity and Compositional Tunability of MXenes

MXenes can be understood as modular two-dimensional nanomaterials whose properties are governed by the coordinated effects of lattice composition, surface chemistry, and interlayer structure. Their general formula, M_n+1_X_n_T_x_, reflects three major design components: the transition-metal layer (M), the carbon and/or nitrogen sublattice (X), and surface terminations (T_x_) such as –O, –OH, –F, or –Cl [19,20]. This structural flexibility allows MXenes to be engineered beyond simple thickness control, enabling regulation of electronic conductivity, hydrophilicity, redox behavior, adsorption capacity, and interfacial compatibility.

**Figure 1:**
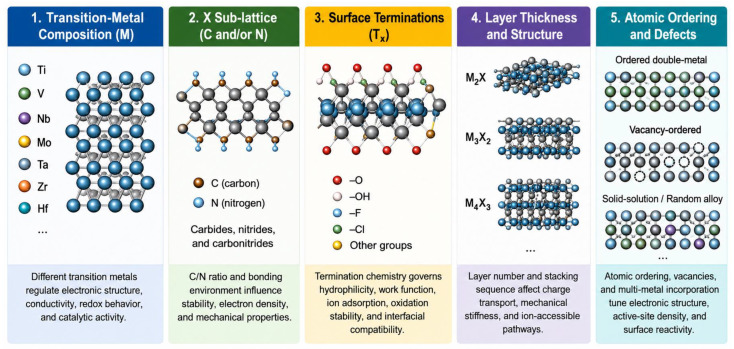
Fundamental structural features and compositional engineering strategies of MXenes.

The M_n+1_X_n_ framework is inherited from layered MAX-phase or related precursors, where strong M–X bonds maintain the carbide or nitride slab after selective removal of the A-layer element [21,22]. Variations in layer thickness influence charge transport, mechanical stiffness, and ion-accessible pathways [23,24]. In addition, the identity and arrangement of transition metals provide important routes for tuning material behavior. Ordered double-transition-metal MXenes, vacancy-ordered MXenes, and solid-solution MXenes demonstrate that atomic ordering, vacancy formation, and multimetal incorporation can be used to adjust electronic structure, active-site density, and surface reactivity [25,26]. The major structural variables that define MXene identity and govern their tunable physicochemical properties are summarized in Figure 1.

Surface terminations are equally important because they directly mediate interactions between MXenes and their surrounding environment. Termination chemistry affects hydrophilicity, work function, ion adsorption, oxidation stability, and compatibility with polymers, oxides, metals, biomolecules, and other two-dimensional materials [27]. Therefore, MXene performance cannot be predicted from nominal composition alone. A rational design approach must consider how lattice composition, atomic ordering, surface termination, defect structure, and interlayer species collectively define structure–property relationships.

### 3.2. Synthesis Routes as Primary Determinants of MXene Structure

Although MXene properties are often discussed after synthesis, the synthesis route itself is one of the most important design variables. Early MXenes were primarily prepared by hydrofluoric acid etching, which selectively removes the A layer from MAX phases and produces multilayered MXene particles with accordion-like morphology [28]. Subsequent intercalation and delamination steps generate few-layer or single-layer nanosheets, but the final material quality depends strongly on etchant concentration, temperature, reaction time, precursor particle size, washing conditions, sonication intensity, and oxidation control [29,30]. These synthesis variables act through several interconnected microscopic pathways. Precursor particle size, phase purity, and crystallinity influence the uniformity and completeness of A-layer removal, whereas etchant identity, concentration, temperature, and reaction duration determine the balance between incomplete etching and excessive structural damage. Insufficient etching may leave residual A elements and poorly separated multilayers, while overly aggressive conditions can increase metal or carbon vacancies, edge damage, surface oxidation, and lattice disorder [29,30]. Etchant composition also influences termination chemistry. Fluoride-containing media generally produce mixed –O, –OH, and –F surfaces, but their relative proportions depend on fluoride activity, acid concentration, water content, temperature, washing history, and subsequent chemical or thermal treatment [27,30]. Intercalant identity and washing conditions further determine the residual-ion content, hydration state, and interlayer environment of the final material.

The development of minimally intensive layer delamination and LiF/HCl etching represented an important advance because it enabled Ti_3_C_2_T_x_ with larger interlayer spacing, improved delamination efficiency, and clay-like processability compared with conventional concentrated HF etching [30,31]. The resulting material can disperse in water without surfactants, form free-standing films, and undergo ion exchange, making it especially useful for scalable electrode, membrane, and coating fabrication [32]. However, LiF/HCl methods also introduce Li^+^ and F-containing terminations, and the relative amounts of –O, –OH, and –F groups remain sensitive to synthesis conditions [27,30]. This is important because surface terminations regulate hydrophilicity, work function, electronic transport, ion adsorption, and chemical stability. Thus, etching should be treated not only as a method for removing the A layer, but also as a surface-chemistry programming step.

**Figure 2:**
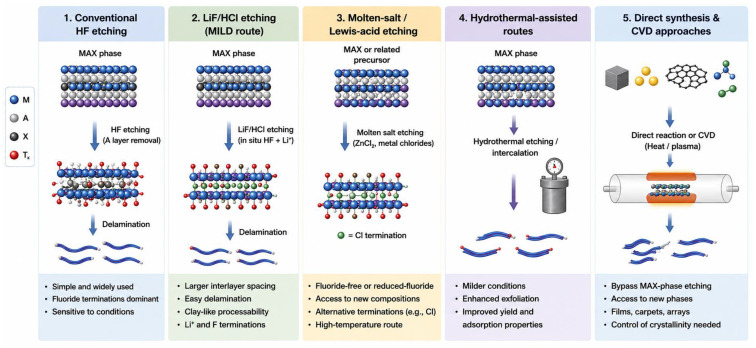
Synthesis-route-dependent engineering of MXene nanomaterials.

Alternative synthesis strategies have been developed to broaden MXene compositions and reduce limitations associated with fluoride-based aqueous etching. Lewis-acid molten-salt etching allows A-site removal from MAX or related precursors using molten salts such as ZnCl_2_ and other metal chlorides, producing MXenes with different surface terminations, including Cl-terminated structures [33,34]. This route is particularly important because it expands the precursor space beyond Al-containing MAX phases and enables access to MXenes that are difficult to obtain through conventional acid etching. Molten-salt methods also provide opportunities to tune surface chemistry under high-temperature conditions, although challenges remain in controlling residual salts, particle morphology, termination uniformity, and scalability [35]. Hydrothermal-assisted etching and intercalation strategies have also been explored to improve exfoliation, yield, and adsorption-related properties, offering milder or more processable alternatives for selected MXene compositions [36,37]. Figure 2 compares the representative synthesis strategies for MXenes, highlighting how different processing routes influence surface chemistry, microstructure, and ultimately the physicochemical properties of the resulting materials.

While these chemical etching and delamination approaches dominate current MXene production, recent efforts have focused on developing direct synthesis routes that expand the compositional space beyond conventional MAX-phase precursors. More recently, direct synthesis and chemical vapor deposition approaches have begun to shift MXene synthesis beyond the classical MAX-phase etching paradigm. Direct reactions of metals, metal halides, graphite, methane, or nitrogen have been used to produce two-dimensional carbide and nitride MXenes, including phases that are not readily accessible from conventional layered precursors [38]. Such approaches are significant because they may reduce dependence on sacrificial A layers and enable new morphologies, thin films, carpets, or spatially controlled structures. Emerging vapor-phase routes for tungsten nitride-based two-dimensional materials further indicate that wafer-scale or substrate-supported MXene-like materials may become increasingly relevant for electronics and advanced devices [39]. Nevertheless, these newer synthesis strategies still require careful evaluation of crystallinity, defect structure, surface chemistry, phase purity, reproducibility, and compatibility with downstream processing.

From a practical engineering perspective, these synthesis routes entail trade-offs among product quality, scalability, operational safety, termination control, and reproducibility. Conventional HF etching remains the most established route and can produce effectively etched multilayer MXenes, particularly Ti_3_C_2_T_x_; however, the use of concentrated HF creates substantial handling and waste-management concerns, and product quality remains sensitive to etchant concentration, reaction time, washing, and delamination conditions [28,29,30]. LiF/HCl etching reduces the need to handle concentrated HF directly and generally facilitates delamination, increases interlayer spacing, and enables the preparation of processable aqueous dispersions. Nevertheless, HF is still generated in situ, and residual Li^+^ together with mixed –O, –OH, and –F terminations can contribute to variability among batches [27,30,31,32]. Molten-salt etching expands the accessible compositional space and enables alternative terminations, particularly Cl-containing surfaces, but its practical implementation is constrained by high processing temperatures, residual-salt removal, morphology control, termination uniformity, and limited scale-up experience [33,34,35]. Hydrothermal-assisted routes may improve etching efficiency, exfoliation, and product yield under relatively processable conditions, although the resulting phase quality, oxidation state, and morphology remain sensitive to temperature, pressure, and reaction duration [36,37]. Direct and vapor-phase synthesis routes avoid conventional A-layer etching and provide opportunities for substrate-supported films and otherwise inaccessible MXene-like phases; however, specialized equipment, phase-purity control, surface-chemistry characterization, and limited batch-level validation currently restricts their scalability and reproducibility [38,39]. Therefore, HF- and LiF/HCl-based routes presently offer the greatest methodological maturity, whereas molten-salt, hydrothermal, and direct routes provide greater compositional or termination flexibility but require further process standardization before reliable large-scale implementation.

### 3.3. Delamination, Interlayer Chemistry, and Solution Processing

After etching, MXene performance is strongly affected by delamination and interlayer engineering. Multilayer MXene powders contain stacked sheets held together by van der Waals interactions, hydrogen bonding, electrostatic interactions, and interlayer species. Intercalants such as dimethyl sulfoxide, tetrabutylammonium hydroxide, metal cations, organic molecules, and water can expand the interlayer spacing and weaken sheet–sheet interactions, enabling exfoliation into few-layer or single-layer nanosheets [40]. Intercalation is not merely a mechanical separation process; it changes ion solvation, surface charge screening, interlayer hydration, and accessibility of active sites. Studies on ion exchange and cation solvation in Ti_3_C_2_T_x_ have shown that interlayer ions and water molecules strongly influence swelling, transport, and electrochemical behavior [32,41]. Therefore, interlayer chemistry is a key bridge between synthesis and functional performance.

Solution processing is one of the major advantages of MXenes compared with many hydrophobic two-dimensional materials. Because Ti_3_C_2_T_x_ and related MXenes possess hydrophilic surface terminations, they can form stable colloidal dispersions in water and selected polar organic solvents [42]. These dispersions enable vacuum filtration, spray coating, spin coating, dip coating, blade coating, printing, and film assembly without requiring harsh binders or surfactants. High-quality Ti_3_C_2_T_x_ films have demonstrated excellent electrical conductivity and optical quality when flake size, oxidation, restacking, and interflake contact are carefully controlled [43,44]. Additive-free MXene inks have also enabled direct printing of microscale devices, indicating that colloidal stability and rheological control are central to scalable manufacturing [45].

Flake size is another important processing parameter. Large flakes generally improve electrical percolation, mechanical integrity, and film conductivity, whereas smaller flakes may provide higher edge density, faster ion accessibility, and better dispersion in composite matrices [46]. Size-dependent studies have shown that lateral dimensions influence optical absorption, electrochemical response, film morphology, and oxidation behavior [46,47]. However, aggressive sonication can introduce defects, reduce conductivity, and accelerate degradation. Therefore, flake-size control must balance exfoliation efficiency with preservation of structural quality. Centrifugation, density-gradient separation, controlled delamination, and careful washing can improve size distribution and reproducibility, but standardized reporting is still needed for meaningful comparison across studies.

A major challenge in MXene processing is their susceptibility to oxidation. In aqueous dispersions, exposure to dissolved oxygen, elevated temperature, light, or unfavorable pH can promote the formation of titanium oxide species, leading to a progressive decline in electrical conductivity [48,49]. The oxidation rate is strongly influenced by synthesis quality, defect density, flake dimensions, concentration, surface chemistry, and storage conditions. Several approaches have therefore been explored to improve stability, including low-temperature storage, oxygen exclusion, antioxidant incorporation, pH adjustment, freeze-drying, solvent exchange, and densification of MXene films [50]. Notably, recent long-term investigations have shown that carefully prepared MXene films can maintain functional stability under ambient conditions, particularly when moisture uptake is minimized. In some cases, conductivity losses associated with environmental exposure can also be partially or fully recovered through drying or thermal annealing [51]. Collectively, these observations suggest that MXene oxidation is not an inherent and unavoidable limitation, but rather a challenge that can be substantially mitigated through controlled synthesis, processing, and storage.

### 3.4. Design Implications for MXene Engineering

The structural and synthetic characteristics outlined above provide a practical basis for the rational engineering of MXenes. Rather than treating synthesis as a fixed sequence of steps, material design should be guided by the requirements of the intended application. This means selecting the composition, MAX-phase precursor, etching chemistry, surface terminations, delamination conditions, flake dimensions, interlayer environment, and assembly method according to the desired structure–property profile [52]. For electrically conductive films and devices, large flakes, low defect densities, efficient interflake contact, and resistance to oxidation are generally preferred. By contrast, applications involving ion storage, separation, or adsorption may benefit from expanded interlayer galleries, accessible surface groups, and a controlled density of defects. In catalysis and sensing, performance is often governed more strongly by surface terminations, edge sites, vacancies, and coordination environments [53]. Accordingly, there is no universally superior synthesis route; the optimal strategy depends on which material attributes are most important for a given function.

In practical terms, the preferred synthesis strategy varies with the intended application. For highly conductive free-standing films, printed electronics, current collectors, and electromagnetic-shielding layers, LiF/HCl etching followed by mild intercalation and delamination is generally advantageous because it can produce large, solution-processable flakes with expanded interlayer spacing and favorable film-forming behavior [30,31,32]. Membrane separation and adsorption applications may similarly benefit from hydrothermal-assisted or intercalation-based routes that enhance hydration, swelling, and access to surface functional groups, provided that interlayer stability and oxidation resistance are maintained. By contrast, catalysis and chemical sensing may benefit from molten-salt, electrochemical, or other termination-engineering routes because alternative surface groups, vacancies, edge sites, and transition-metal coordination environments can provide more tunable adsorption and reaction sites [33,34,35,53]. For substrate-integrated electronics and spatially defined devices, direct or vapor-phase synthesis is particularly attractive because it can generate supported films without conventional exfoliation and reassembly, although phase purity, equipment requirements, and scalability remain limiting [38,39]. Finally, applications involving biological contact or environmental release should prioritize fluoride-reduced or fluoride-free preparation, thorough removal of residual reagents, and low-oxidation processing; however, these routes must still demonstrate reproducibility and product quality comparable with established HF- or LiF/HCl-based methods.

A meaningful structure–property analysis of MXenes must therefore go beyond cataloguing compositions and applications. The central issue is how individual design variables influence the resulting material. The identity of the transition metal determines key aspects of the electronic structure and redox behavior, whereas the carbon or nitrogen sublattice affects bonding, conductivity, and chemical stability [54]. Layer thickness and flake size influence electron and ion transport, while surface terminations regulate wettability, interfacial interactions, and chemical reactivity. Intercalated species control swelling, ion diffusion, and interlayer accessibility, and defects can generate active sites at the expense of structural or oxidative stability [55]. Processing history further shapes dispersion quality, film morphology, contact resistance, and batch-to-batch reproducibility. Clarifying these relationships is essential for moving MXenes beyond proof-of-concept studies and toward reliable, application-ready materials [56]. The following sections, therefore, examine how these interconnected design parameters govern MXene properties, functionalization, and performance across different technological contexts. The major synthesis and processing routes are summarized in Table 1, with emphasis on their effects on product quality, termination chemistry, process scalability, operational safety, and batch-to-batch reproducibility.

## 4. Structure–Property Relationships and Functional Properties of MXene Nanomaterials

### 4.1. Electronic Structure, Conductivity, and Interfacial Charge Behavior

The functional performance of MXenes is strongly rooted in their electronic structure. Many pristine transition-metal carbide and nitride MXenes are predicted to exhibit metallic conductivity because transition-metal d orbitals contribute substantially to the electronic density of states near the Fermi level [62]. This feature differentiates MXenes from many semiconducting two-dimensional materials and provides an important foundation for their use in conductive films, electrodes, electromagnetic shielding layers, sensors, and current collectors. At the atomic level, the identity of the M element controls the number, energy, and spatial extent of the d states that dominate the electronic structure near the Fermi level. Across the early transition-metal series, changes in nominal d-electron filling shift the Fermi level through M–X-hybridized bonding, nonbonding, and antibonding states, thereby modifying carrier density, band dispersion, accessible oxidation states, and the occupancy of orbitals involved in surface bonding. The relatively localized 3d orbitals can form narrower bands and may be more sensitive to exchange and on-site electron-correlation effects, whereas the more spatially extended 4d and 5d orbitals generally produce stronger M–X and M–M overlap and broader electronic bands. For heavier 5d elements, spin–orbit coupling may additionally influence band splitting and electronic topology. Consequently, replacing Ti with V, Nb, Mo, Ta, W, or a combination of metals can alter conductivity, redox activity, adsorption strength, and catalytic-intermediate binding through coupled changes in the density of states at the Fermi level, d-band position, bond covalency, and interfacial charge transfer [26,62]. These trends are experimentally illustrated by Ti-, Nb-, and V-containing M-site solid-solution MXenes, for which variation in metal composition enabled the macroscopic conductivity to be tuned over approximately three orders of magnitude at room temperature [26].

However, electronic behavior is not determined by the carbide or nitride lattice alone. Surface terminations can significantly alter band dispersion, work function, charge density, and interfacial dipole formation [63,64]. For example, theoretical studies have shown that –OH-terminated MXenes may exhibit ultralow work functions, whereas –O and –F terminations can shift electronic structure in different directions depending on the transition-metal composition [65]. Therefore, termination chemistry should be considered an active electronic design parameter rather than a passive residue of synthesis.

Defects, vacancies, and mixed terminations further complicate MXene electronic properties. Atomic-scale defects in Ti_3_C_2_T_x_ can introduce local structural distortions, modify charge distribution, and affect carrier scattering [66]. Meanwhile, mixed –O, –OH, and –F terminations may create heterogeneous surface potentials that influence adsorption, electron transfer, and electrochemical response [35,55]. Experimental spectroscopic studies have confirmed that local bonding in Ti_3_C_2_T_x_ depends on Ti–C bonding, Ti–O/F coordination, and the spatial organization of termination species [67,68]. These findings explain why MXenes with the same nominal formula may show different conductivity, sensing response, or catalytic behavior when synthesized under different conditions. Thus, electronic structure should be interpreted as a result of coupled lattice composition, defect chemistry, and surface bonding. Accurate interpretation is complicated by the difficulty of producing and characterizing a single termination species. Wet-chemical synthesis usually generates spatially heterogeneous mixtures of –O, –OH, and –F together with intercalated water, residual ions, adsorbed species, and, in some cases, surface oxides. X-ray photoelectron spectroscopy provides useful average compositional information, but assignment and quantification of individual oxygen-containing species may be complicated by overlapping contributions from –O, –OH, water, and oxidation products. Solid-state ^1^H and ^19^F NMR can provide complementary information on hydroxyl and fluorine environments; for example, NMR analysis of Ti_3_C_2_T_x_ demonstrated that –F and –OH groups were intimately mixed and that their relative proportions depended strongly on the synthesis route [27]. Spectroscopic methods should therefore be combined with structural and chemical analyses rather than using a single fitted XPS spectrum as definitive evidence of termination identity.

Interfacial charge behavior is equally important because MXenes rarely function as isolated monolayers in practical devices. In films, membranes, and composites, charge transport depends on both intraflake conductivity and interflake junction resistance [69]. Iravani et al. (2024) reviewed how artificial intelligence can accelerate MXene research by predicting material properties, optimizing synthesis, and guiding applications [70]. It highlights MXenes’ potential in energy storage, sensing, catalysis, actuators, and neuromorphic systems. Data-AI-driven models can connect structural features with performance, streamline experimental design, and identify promising compositions, although limited datasets and complex structure–property relationships remain major challenges for future development [70,71]. Flake size introduces a direct trade-off between electronic continuity and electrochemical accessibility. Maleski et al. (2018) fractionated Ti_3_C_2_T_x_ flakes over a lateral-size range of approximately 0.1–5 μm and found that films assembled from flakes of approximately 1 μm provided the most favorable electrochemical balance, delivering 290 F g^−1^ at 2 mV s^−1^ and retaining 200 F g^−1^ at 1000 mV s^−1^ [46]. Larger flakes generally decrease the number of resistive interflake junctions and favor macroscopic conductivity, whereas excessively small flakes increase edge density and ion-accessible pathways but may also introduce additional boundaries and sonication-induced defects. Flake dimensions should therefore be optimized according to the relative importance of electron transport, ion accessibility, film integrity, and oxidation resistance rather than treated as a parameter for unidirectional minimization or maximization. Surface water, adsorbed gases, and environmental molecules can also alter charge transport by interacting with termination groups and changing local carrier density [51,72]. This sensitivity is beneficial for sensing but problematic for long-term electronic stability. Conductivity optimization requires simultaneous control of flake quality, termination chemistry, film assembly, interflake contact, and environmental exposure.

These electronic principles provide a basis for predicting M-dependent performance, although neither elemental identity nor nominal d-electron count is sufficient as an isolated descriptor. First-principles calculations can screen candidate MXenes using physically meaningful quantities, such as the density of states near the Fermi level, band dispersion, effective carrier mass, d-band center, charge-transfer tendency, adsorption energy, accessible oxidation states, and M–X formation energy. Correlation-sensitive methods, for example, DFT + U or related approaches, may be required for systems containing relatively localized 3d states, whereas spin–orbit coupling should be considered for heavier 5d elements. These calculated descriptors can subsequently be combined with elemental variables, including group number, electronegativity, atomic radius, and nominal d-electron filling, in machine-learning and inverse-design models. Nevertheless, reliable prediction requires the simultaneous inclusion of the M and X elements, surface terminations, layer thickness, vacancies, atomic ordering, interlayer species, and assembly architecture because each of these variables can reconstruct the same d-derived electronic states. Predictive screening should therefore be used to prioritize experimentally testable MXene compositions rather than to assign performance solely from periodic-table trends.

### 4.2. Surface Chemistry, Redox Activity, and Ion-Transport Properties

Unlike purely double-layer carbon materials, MXenes can store charge through surface-controlled redox reactions, cation intercalation, and pseudocapacitive processes [73,74]. Sodium-ion intercalation studies have shown that ions can enter the MXene interlayer space while maintaining relatively fast kinetics [75]. Similarly, high-power sodium-ion hybrid capacitor studies demonstrated that MXene nanosheets can support rapid pseudocapacitive charge storage [73]. These behaviors arise from the coexistence of conductive transition-metal carbide layers, hydrophilic terminations, and interlayer pathways that accommodate solvated or partially desolvated ions. Surface terminations participate directly in these charge-storage processes rather than serving only as passive wetting groups. Oxygen-containing terminations can support proton-coupled electron transfer and reversible changes in the oxidation state of surface transition-metal atoms, whereas –F groups are generally less active for proton storage and may occupy sites that would otherwise contribute to redox reactions or ion adsorption.

**Figure 3:**
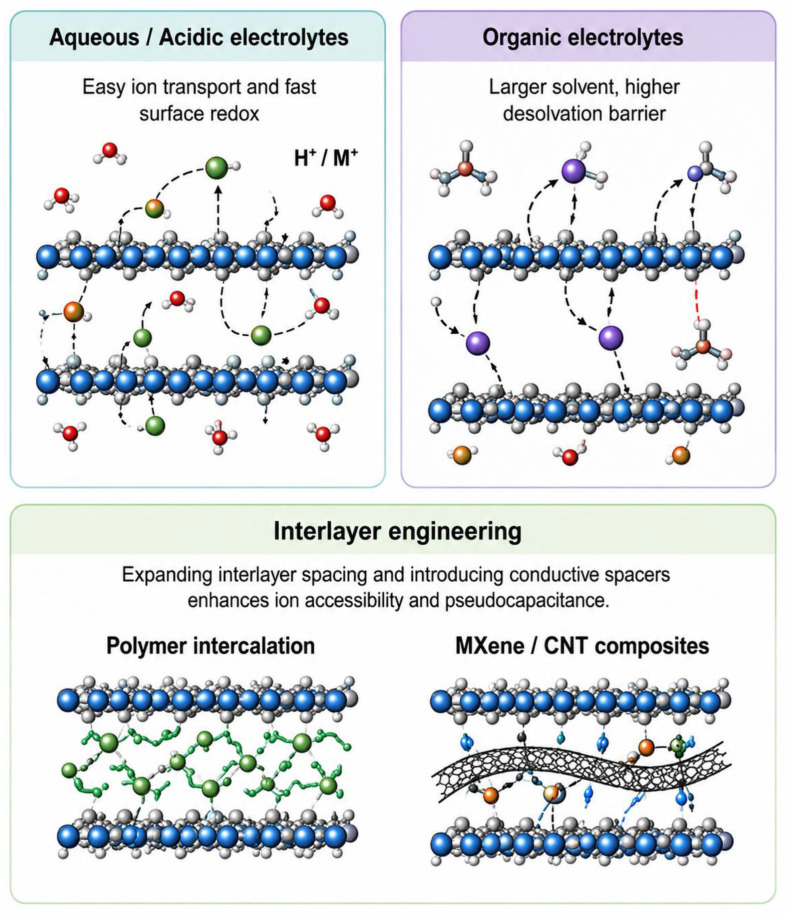
Electrolyte-Dependent Ion Transport and Interlayer Engineering Strategies for Enhancing Electrochemical Performance of MXenes.

The electrochemical properties of MXenes are highly sensitive to electrolyte composition and solvent environment. Solvent-dependent charge-storage studies revealed that ion desolvation and confinement within Ti_3_C_2_T_x_ interlayers strongly influence capacitance and kinetics [76]. In acidic and aqueous electrolytes, proton and cation transport can be coupled with fast surface redox reactions, while in organic electrolytes, solvent size and desolvation barriers can limit access to interlayer active sites [77]. Polymer intercalation and conductive polymer growth between MXene sheets can further modify redox behavior by expanding the interlayer spacing and introducing additional pseudocapacitive components [78]. MXene/carbon nanotube composite papers also illustrate how conductive spacers can reduce restacking, improve ion accessibility, and maintain high volumetric capacitance [79]. A direct example is provided by LiF/HCl-derived Ti_3_C_2_T_x_ clay, in which hydration-induced swelling and improved accessibility of the interlayer galleries enabled additive-free films to reach a volumetric capacitance of up to 900 F cm^−3^, nearly twice that reported previously for conventionally prepared Ti_3_C_2_T_x_ electrodes [31]. This improvement demonstrates that interlayer expansion can increase electrolyte penetration and utilization of redox-active surfaces. Nevertheless, greater spacing is not invariably advantageous because excessive expansion or spacer incorporation may reduce electrode packing density, volumetric performance, and interflake electronic contact. The relevant design parameter is, therefore, accessible and stable interlayer volume rather than only interlayer distance. Figure 3 summarizes how electrolyte selection and interlayer engineering jointly regulate ion transport, charge-storage mechanisms, and electrochemical performance in MXenes. Specifically, aqueous and acidic electrolytes facilitate rapid ion transport and surface redox reactions, whereas organic electrolytes exhibit slower ion diffusion because of larger solvated ions.

Ion transport is governed not only by interlayer spacing but also by surface charge, hydration structure, cation size, and termination chemistry. MXene membranes can exhibit charge- and size-selective ion sieving because interlayer galleries act as confined transport channels [80]. Ultrafast water transport through MXene membranes further demonstrates that hydrophilic surface groups and ordered nanochannels can promote rapid molecular permeation while still allowing selective separation [81]. These transport behaviors are important because they reveal a broader structure–property principle: MXene interlayers are not empty spaces but dynamic environments containing water, ions, functional groups, and electrostatic interactions. Consequently, rational MXene design requires control over both basal-plane chemistry and confined interlayer chemistry.

### 4.3. Mechanical, Optical, Electromagnetic, and Stability-Related Properties

In addition to electronic and electrochemical properties, MXenes exhibit important mechanical and optical characteristics that depend strongly on flake assembly and interfacial bonding. Individual flakes possess high in-plane stiffness, while assembled films derive their macroscopic strength from flake alignment, interlayer interactions, hydrogen bonding, surface terminations, and the presence of bridging agents [82]. Elastic measurements of monolayers and bilayers confirmed that MXenes can retain strong mechanical properties at the nanoscale [83]. However, free-standing films may be mechanically limited by weak interflake adhesion. Bridging-induced densification strategies have shown that strengthening interflake interactions can produce scalable MXene films with substantially improved tensile strength and toughness [84]. Similarly, MXene/polymer nanocomposites demonstrate that polymer chains can improve flexibility and processability while maintaining electrical conductivity when interfacial compatibility is properly controlled [85].

The relationship between flake assembly and macroscopic performance is demonstrated by sequentially densified Ti_3_C_2_T_x_ films prepared through small-flake intercalation followed by Ca^2+^/borate interfacial bridging. The resulting films reached a tensile strength of 739 MPa, a Young’s modulus of 72.4 GPa, and an electrical conductivity of 10,336 S cm^−1^ [82]. Before ionic bridging, the incorporation of an optimized 10 wt% fraction of small flakes increased tensile strength from approximately 185 MPa for large-flake films to 409 MPa by filling interflake voids, while maintaining conductivity above 10,000 S cm^−1^. This example demonstrates that controlled mixed-flake assembly and interlayer bonding can improve mechanical load transfer without necessarily sacrificing electronic transport. However, excessive small-flake incorporation decreases alignment and introduces additional boundaries, again emphasizing the existence of an optimum structural configuration rather than a monotonic size effect.

Optical and electromagnetic properties are also closely linked to electronic structure and film morphology. Highly conductive, solution-processed Ti_3_C_2_T_x_ films can exhibit optical transparency at low thickness while maintaining electrical conductivity, making them relevant to transparent electrodes and optoelectronic interfaces [86]. MXene films also show plasmonic behavior, strong broadband light absorption, and efficient light-to-heat conversion due to high free-carrier density and metallic character [87,88]. These properties can be tuned through thickness, flake orientation, surface oxidation, and film density. In the electromagnetic domain, MXenes are effective shielding and absorption materials because conductive networks promote reflection, absorption, and multiple internal scattering [89]. However, high conductivity alone is not sufficient; impedance matching, film thickness, porosity, flake alignment, and interfacial polarization also determine electromagnetic attenuation behavior.

MXene surfaces are highly responsive to adsorbed molecules, which can be beneficial for sensing but challenging for stability. Metallic gas sensors have shown strong signal-to-noise responses because gas adsorption can alter interfacial charge transport and surface carrier density [90,91]. However, the same chemical reactivity that supports sensing also makes MXenes vulnerable to oxidation, hydrolysis, and environmental degradation. Oxidation can reduce conductivity, generate metal oxide species, disrupt flake integrity, and change surface chemistry. Stability is influenced by synthesis quality, defect density, flake size, water content, dissolved oxygen, storage temperature, pH, and film morphology [51,72]. Importantly, recent long-term studies suggest that well-prepared MXene films can maintain useful environmental stability over extended periods, and that conductivity losses may partly arise from reversible water uptake rather than irreversible structural degradation [51]. This distinction is important because it shifts the discussion of stability from a simple weakness in MXenes to a controllable structure–processing issue. Accordingly, transition-metal selection should be understood as electronic-structure engineering rather than simple elemental substitution, because changes in d-state filling and hybridization propagate into conductivity, redox activity, adsorption, and catalytic performance. The main structural variables that control MXene functional properties are summarized in Table 2, emphasizing that MXene performance is governed by coupled lattice, surface, interlayer, and assembly-level factors.

Taken together, MXene properties arise from the interaction of multiple structural levels: atomic composition, termination chemistry, defect density, interlayer environment, flake morphology, and assembled architecture. A material optimized for one property may not be optimal for another. High conductivity may require large flakes and dense films, whereas fast ion transport may require open interlayers and reduced restacking. Strong mechanical films need interflake adhesion, while sensing platforms may benefit from high surface accessibility and environmental responsiveness. Therefore, structure–property relationships provide the central logic for MXene functional design. The next section builds on these property principles by examining how functionalization, heterostructure construction, and composite engineering are used to tune MXenes for advanced technologies deliberately.

## 5. Emerging Applications of Engineered MXene Nanomaterials

### 5.1. Energy Storage and Electrochemical Devices

Energy storage remains one of the most extensively explored application areas for MXene nanomaterials because MXenes combine metallic conductivity, hydrophilic surfaces, redox-active transition-metal sites, and accessible interlayer galleries. Early studies demonstrated that Ti-, V-, Nb-, and Mo-based MXenes could serve as promising electrodes for lithium-ion, sodium-ion, potassium-ion, and multivalent metal-ion batteries [100,101]. In these systems, charge storage is governed by ion intercalation, surface redox reactions, electrolyte accessibility, and the ability of the MXene layers to maintain electrical continuity during repeated cycling. Ti_3_C_2_T_x_ has been particularly attractive because its high conductivity and solution processability allow the fabrication of free-standing electrodes, conductive binders, current collectors, and hybrid architectures [102].

For supercapacitors, MXenes provide high volumetric capacitance because dense films can retain excellent electrical conductivity while supporting fast ion transport [78,103]. However, restacking of nanosheets can restrict electrolyte access and reduce rate performance. To address this limitation, researchers have introduced conductive spacers, porous architectures, polymer interlayers, carbon nanotubes, graphene, metal oxides, and pillaring agents to expand interlayer spacing and improve ion diffusion [104,105]. For example, MXene/carbon nanotube papers, MXene/polyaniline hybrids, and three-dimensional porous MXene frameworks have shown improved capacitance retention and rate capability compared with densely restacked MXene films [106]. These examples illustrate that high conductivity alone is insufficient; successful electrochemical design requires balancing electron transport, ion accessibility, mechanical integrity, and interlayer stability.

MXenes have also been investigated as functional components in lithium–sulfur batteries, zinc-ion batteries, and hybrid capacitors. In lithium–sulfur systems, polar MXene surfaces can help immobilize lithium polysulfides, improve sulfur utilization, and suppress shuttle effects [107,108]. In aqueous zinc-ion batteries, halogen-terminated MXenes and MXene-based cathodes have shown that surface chemistry can directly contribute to electrochemical activity rather than merely serving as a structural support [88]. Beyond electrodes, MXenes are increasingly used as conductive scaffolds, protective interlayers, separators, and current collectors, indicating their versatility across multiple device components [81,109]. The major design principle is clear: MXene-based energy devices perform best when surface chemistry, interlayer spacing, flake assembly, and electrolyte compatibility are engineered together.

### 5.2. Environmental Remediation, Membranes, and Molecular Separation

MXene materials are also promising for environmental remediation as their hydrophilic surfaces, high surface area, tunable charge, and abundant functional groups support adsorption, reduction, ion sieving, and membrane-based separation. Early work showed that two-dimensional titanium carbide could adsorb and decompose organic dyes in aqueous media, indicating its potential for pollutant removal and catalytic environmental treatment [105]. MXenes have also shown strong affinity for metal ions due to surface –O, –OH, and related functional groups. For example, activated hydroxyl groups on titanium carbide were reported to enable unusual lead adsorption behavior, while Ti_3_C_2_-based materials have been used for reductive removal of highly toxic Cr(VI) from water [104,110]. These studies demonstrate that surface terminations are central to environmental performance because they regulate electrostatic attraction, complexation, redox interaction, and adsorption selectivity.

Membrane-based applications expand this concept from dispersed adsorbents to structured separation platforms. Lamellar MXene membranes have been developed for water purification, where stacked nanosheets create confined nanochannels for selective transport [107]. MXene membranes have also been investigated for desalination, pervaporation, gas separation, and ion sieving, with performance governed by interlayer spacing, surface charge, hydration structure, and nanosheet alignment [80,81]. In flow-electrode capacitive deionization, Ti_3_C_2_T_x_ MXene slurry electrodes were used for removal and recovery of ammonia from simulated wastewater, showing that high conductivity and ion adsorption capacity can improve electrochemical separation efficiency [109]. Such systems are especially attractive for nutrient recovery and circular water treatment because the target species can be removed and potentially regenerated rather than simply transferred to secondary waste.

MXenes have been further incorporated into antimicrobial and antifouling membranes. The nanosheet-based membranes exhibited antibacterial activity, suggesting potential value for water treatment systems where microbial fouling reduces membrane lifetime [111]. Composite membranes containing MXenes and polymers have been designed to improve mechanical integrity, fouling resistance, and molecular selectivity [112]. However, environmental applications require careful evaluation of MXene oxidation, nanoparticle release, long-term membrane stability, regeneration chemistry, and potential ecotoxicity. Thus, the most promising environmental platforms are likely to be immobilized MXene membranes, composites, or recoverable electrode systems rather than freely dispersed nanosheets.

### 5.3. Sensors, Flexible Electronics, Electromagnetic Devices, and Smart Interfaces

MXenes are highly attractive for sensors and flexible electronics because they combine high conductivity, solution processability, large surface area, and chemically active surfaces. Surface-enhanced Raman scattering (SERS) represents one important example. MXenes have been reported as a SERS substrate capable of enhancing molecular signals through charge-transfer interactions and surface adsorption [113]. More advanced MXene-based SERS platforms have been used for selective detection of salicylic acid and biomolecular targets, including virus-related proteins, by combining electromagnetic and chemical enhancement mechanisms [114,115]. In electrochemical sensing, Ti_3_C_2_-based interfaces have been used to immobilize biomolecules and promote direct electron transfer, enabling biosensors for nitrite, glucose-related systems, and other analytes [116,117]. MXene-based wearable biosensor systems have also been developed for sweat analysis, showing the potential of MXenes in personalized health monitoring and flexible diagnostic platforms [118].

Gas sensing is another strong application because adsorbed molecules can alter charge transport in MXene films. Ti_3_C_2_T_x_ gas sensors have shown high sensitivity toward gases such as NH_3_, NO_2_, acetone, ethanol, and volatile organic compounds, with sensing behavior influenced by surface terminations, humidity, oxidation state, and interflake junctions [90,91]. For flexible strain and pressure sensors, MXenes are often integrated with elastomers, textiles, hydrogels, or porous substrates to convert mechanical deformation into resistance changes [119]. These devices benefit from MXene conductivity and percolated network formation, but long-term reliability depends on controlling flake adhesion, crack formation, hydration, and oxidation during repeated bending or stretching.

Electromagnetic and wireless technologies represent another rapidly growing field. MXenes have been used in printed antennas, radio-frequency devices, transparent conductive films, electrochromic devices, and electromagnetic interference shielding materials [120]. Solution-processed Ti_3_C_2_T_x_ antennas demonstrated that MXene inks can be printed into functional communication components, while inkjet-printed MXene/protein electrodes enabled stimuli-responsive electromagnetic shielding [108,113]. MXene-based triboelectric nanogenerators and photonic devices further illustrate how conductivity, surface charge, optical absorption, and mechanical flexibility can be combined in smart interfaces [121]. These examples highlight a broader design principle: for electronic and electromagnetic applications, MXene performance is governed not only by intrinsic conductivity but also by film uniformity, ink rheology, flake orientation, interflake contact, substrate compatibility, and environmental stability.

### 5.4. Biomedical, Antimicrobial, and Photothermal Technologies

Biomedical and antimicrobial applications of MXenes have expanded because of their strong near-infrared absorption, photothermal conversion, large surface area, and capacity for surface functionalization. Ti_3_C_2_ was reported as an efficient light-to-heat conversion material, while ultrathin MXene ceramic nanosheets demonstrated strong photothermal performance under near-infrared irradiation [88]. Niobium carbide MXene was further developed for photothermal tumor eradication in both NIR-I and NIR-II biological windows, showing that MXene composition can tune optical and biological performance [122]. In addition, MXene quantum dots and surface-modified MXenes have been explored for bioimaging, drug delivery, and photothermal therapy, where size, surface charge, dispersibility, and degradation behavior strongly influence biological response [123,124].

MXenes have also shown antimicrobial potential. Ti_3_C_2_T_x_ MXene exhibited antibacterial activity against Gram-negative and Gram-positive bacteria, with mechanisms attributed to membrane interaction, physical disruption, oxidative stress, and contact-mediated effects [125]. Antibacterial MXene membranes provide an applied route where antimicrobial activity is combined with filtration or surface protection [111]. More recent studies have explored MXene-based photothermal antibacterial platforms and wound-related disinfection systems, where localized heating can enhance bacterial killing while reducing chemical antimicrobial use [126]. However, biomedical and antimicrobial uses require stricter safety assessment than many electronic or catalytic applications. Cytotoxicity, hemocompatibility, immune response, biodistribution, degradation products, and long-term clearance must be evaluated before translation [127].

Across these application areas, MXene technologies are moving from simple material demonstration toward system-level engineering. In energy devices, the key challenge is balancing ion accessibility with electrode density and cycling stability. In environmental systems, adsorption and separation must be combined with regeneration and release control. In sensors and electronics, high sensitivity must be balanced with device stability under humidity, strain, and oxidation. In biomedical systems, photothermal and antimicrobial performance must be integrated with biocompatibility and degradability. Therefore, emerging MXene applications require application-specific design rather than a universal material strategy. The most successful MXene platforms will be those in which composition, surface chemistry, morphology, interface, and device architecture are co-designed for the intended operating environment. Representative application examples are summarized in Table 3 to highlight how MXene composition, surface chemistry, architecture, and interfacial design enable diverse emerging technologies.

### 5.5. Recent Advances in MXene Applications for Food Systems

MXene-based electrochemical and optical sensors have been developed for monitoring, detecting, and sterilizing pesticides, antibiotics, mycotoxins, heavy metals, unauthorized additives, spoilage indicators, and foodborne microorganisms [132]. For example, an electrospun Ti_3_C_2_T_x_ MXene/polyvinylidene fluoride nanofiber aptasensor provided a sensitive platform for detecting *ochratoxin* A, demonstrating the suitability of MXenes for mycotoxin screening in food matrices [133]. Similarly, a Ti_3_C_2_T_x_/AuPt/acetylcholinesterase electrochemical platform enhanced electron transfer and catalytic activity for sensitive chlorpyrifos detection [134]. Beyond sensing, MXenes can function as antimicrobial components in active food-contact materials. Santos et al. incorporated Ti_3_C_2_T_x_ into polylactic acid surfaces and reported strong bactericidal activity against *Listeria monocytogenes* and *Salmonella enterica*, supporting its application in antimicrobial food packaging [135]. More recently, Ag nanoparticles grown on Ti_3_CNT_x_ MXene improved antibacterial performance against Gram-positive and Gram-negative bacteria and showed potential for incorporation into paper-based packaging materials [136]. MXenes may also enhance packaging barrier strength, mechanical integrity, and responsiveness to environmental or spoilage-related changes. Makani et al. (2025) developed a Ti_3_C_2_T_x_ MXene quantum dot-based electrochemical cholinesterase biosensor for organophosphorus pesticide detection [137]. The sensor achieved an ultralow detection limit (1 × 10^−17^ M) and excellent selectivity toward chlorpyrifos, acephate, and glyphosate, demonstrating rapid, sensitive, and reliable performance for environmental and food safety monitoring. Nevertheless, their practical translation requires careful evaluation of oxidation stability, nanoparticle migration, residual etching chemicals, cytotoxicity, environmental fate, and regulatory compliance. Future research should emphasize food-simulant migration studies, standardized toxicological assessment, scalable fluorine-free synthesis, and validation in complex foods under realistic processing and storage conditions.

## 6. Translational Challenges, Safe Design, and Future Perspectives

### 6.1. Stability, Reproducibility, and Structure Control

Despite rapid progress, the practical translation of MXene nanomaterials remains limited by interconnected challenges related to oxidation, storage stability, reproducibility, and structural preservation. Oxidation behavior should be interpreted according to the physical form in which MXenes are stored and used. Aqueous dispersions are generally the most oxidation-sensitive form because water and dissolved oxygen have continuous access to the large exposed surface area of dispersed nanosheets. Their degradation rate is strongly influenced by temperature, light exposure, pH, flake dimensions, defect density, residual etchants, surface chemistry, concentration, and storage conditions, with oxidation leading to transition-metal oxide formation, altered surface terminations, and progressive conductivity loss [138,139,140]. Dried MXene powders eliminate continuous contact with dissolved oxygen but should not be assumed to be intrinsically stable. Residual water, humid storage, high defect density, and incomplete removal of synthesis residues can continue to promote oxidation. Drying may also induce irreversible restacking, which can preserve the apparent solid material while reducing its subsequent redispersibility and accessible surface area. Free-standing films frequently exhibit slower degradation than dilute dispersions because compact flake assembly limits oxygen and moisture transport into the film interior [141,142]. Nevertheless, oxidation may initiate at exposed edges, cracks, defects, and poorly contacted regions. Moisture uptake may also alter film conductivity without necessarily causing complete structural decomposition. Carefully prepared and densified MXene films can therefore retain functional stability under ambient conditions, and some conductivity losses may be partially recovered through drying or thermal annealing [50,51].

Composite materials represent a further distinct case. Embedding MXenes within polymeric, ceramic, carbonaceous, or hybrid matrices can immobilize the nanosheets and reduce their direct exposure to oxygen and water. However, the degree of protection depends on matrix permeability, interfacial adhesion, MXene dispersion, filler loading, porosity, and processing history. Dense and hydrophobic matrices may provide effective environmental barriers, whereas water-rich, porous, or poorly bonded matrices may offer limited protection. Moreover, matrix encapsulation can improve oxidation resistance while simultaneously restricting ion transport, adsorption, catalytic-site accessibility, or sensing response. Composite stability should therefore be evaluated for the complete material system rather than inferred from the stability of either the MXene or matrix alone.

Reproducibility is another critical issue because nominal composition does not uniquely define the structural state of a MXene. Variations in MAX-phase purity and particle size affect etching uniformity and residual A-element content; etchant composition, concentration, temperature, and duration influence vacancy formation and termination chemistry; washing and intercalation determine residual ions, hydration, and interlayer spacing; and delamination, sonication, and centrifugation regulate lateral-size distribution and edge-defect density [29,143]. Storage history can then further modify oxidation state, surface composition, and colloidal stability. Thus, apparently equivalent M_n+1_X_n_T_x_ samples may possess substantially different defect densities, flake dimensions, termination ratios, interlayer species, and oxidation levels. These variations affect macroscopic properties through distinct mechanisms. Increased vacancy and edge-site density may enhance adsorption, catalysis, or pseudocapacitive activity but can also increase carrier scattering and oxidative degradation. Larger flakes generally improve electrical percolation and mechanical load transfer, whereas smaller flakes facilitate dispersion and ion accessibility but create more interflake junctions. Differences in –O, –OH, and –F ratios alter surface dipoles, work function, wettability, redox activity, and interfacial bonding. Reproducibility therefore requires process-specific quality control rather than reliance on nominal composition alone. At minimum, studies should report precursor characteristics, reagent concentrations, solid-to-liquid ratio, reaction temperature and duration, washing endpoint, delamination and sonication energy, centrifugation conditions, flake-size distribution, defect indicators, quantitative termination composition, residual ions, water content, oxidation state, and storage history.

From a translational perspective, the successful implementation of MXene technologies requires addressing multiple materials and engineering challenges. These key issues and their potential mitigation strategies are summarized in Table 4.

Future MXene research should therefore adopt more standardized reporting of precursor identity, etching conditions, termination chemistry, elemental composition, flake dimensions, zeta potential, oxidation state, residual ions, water content, and storage conditions.

### 6.2. Scalable Processing and Device Integration

The transition from laboratory-scale MXene demonstrations to industrial technologies requires scalable and reproducible processing. MXenes are attractive because they can be dispersed in water and processed into films, coatings, membranes, fibers, aerogels, hydrogels, inks, and composites using solution-based methods [45,89]. Printing and coating approaches are especially promising for flexible electronics, electromagnetic shielding, sensors, antennas, and wearable devices because they allow patterned deposition on plastic, textile, paper, glass, or elastomeric substrates [45,89]. However, large-scale processing introduces additional constraints, including ink rheology, shelf life, flake alignment, drying stress, adhesion to substrates, film cracking, oxidation during processing, and compatibility with existing manufacturing lines.

**Figure 4:**
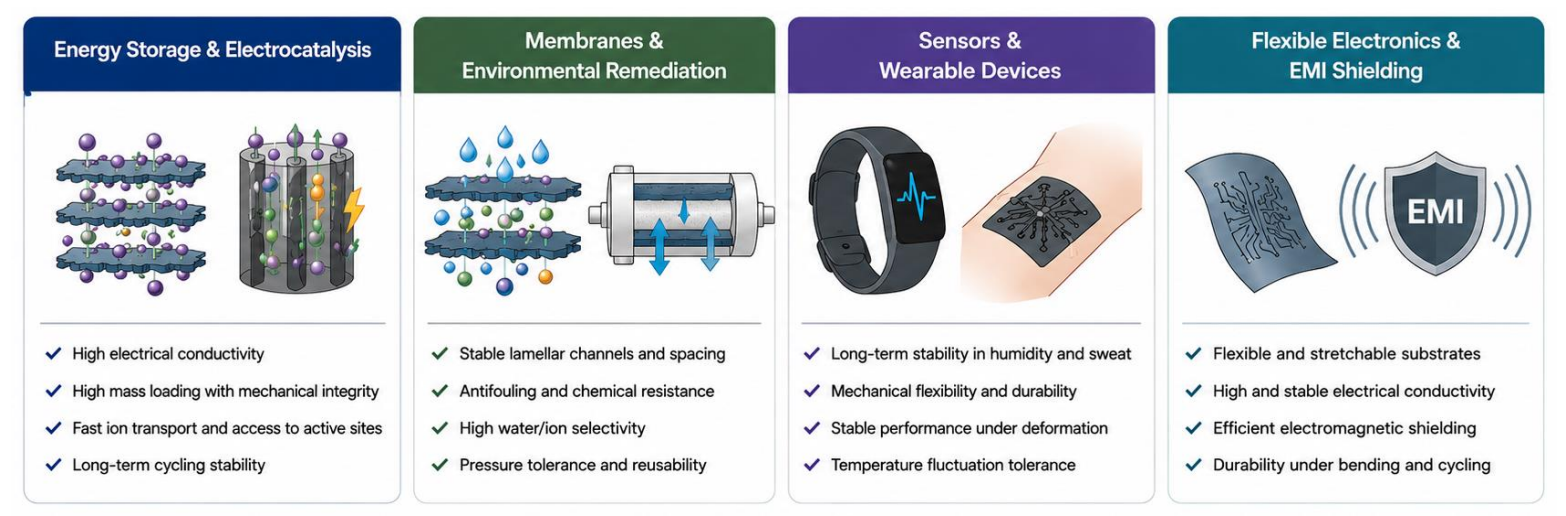
Application-specific engineering requirements for scalable MXene technologies.

For energy storage and electrocatalysis, scalable MXene electrodes must balance conductivity, ion accessibility, mass loading, mechanical integrity, and cycling stability [77,100]. High-density films are valuable for volumetric capacitance, whereas porous or vertically aligned architectures can improve ion transport at high rates [77,100]. For membranes and environmental remediation, scale-up requires stable lamellar channels, controlled swelling, antifouling resistance, pressure tolerance, and regeneration capability [149]. For sensors and wearable devices, long-term operation under humidity, sweat, mechanical deformation, and temperature fluctuation must be evaluated [118]. These application-specific requirements indicate that industrial MXene development cannot rely only on high intrinsic material performance; it must integrate materials synthesis with device engineering and real operating conditions. Figure 4 summarizes the key engineering considerations required to translate MXene materials into scalable, application-specific technologies.

Another important translation issue is the compatibility of MXenes with polymeric, ceramic, metallic, and biological interfaces. MXenes are frequently incorporated into polymers to improve electrical conductivity, electromagnetic shielding, mechanical reinforcement, thermal management, or sensing response [150]. However, dispersion quality, interfacial adhesion, polymer crystallinity, filler orientation, and percolation behavior strongly influence final composite performance. Poor dispersion can reduce mechanical strength, create defects, or accelerate degradation, while excessive MXene loading can compromise flexibility, transparency, or processability. Therefore, future composite design should focus not only on adding MXenes but on controlling interfacial chemistry, nanosheet orientation, matrix compatibility, and long-term durability.

### 6.3. Safety, Sustainability, and Responsible Development

As MXene applications expand into water treatment, biomedical systems, food-contact technologies, wearable devices, and environmental platforms, safety and sustainability must be considered more systematically. Current toxicological studies suggest that biological responses depend on MXene composition, flake size, surface terminations, oxidation state, dose, exposure route, and degradation products [151]. Ti_3_C_2_T_x_, Nb_2_C, Mo_2_C, and other MXenes may differ substantially in cytotoxicity, oxidative stress response, membrane interaction, immune effects, and biodegradation behavior [127,152]. Therefore, safety conclusions from one MXene composition or exposure model should not be generalized across the entire MXene family.

The drive for enhanced stability and targeted functionality has led to the development of MXene-zeolitic imidazolate framework (MXene@ZIF-8) composites, which broaden application scopes in water purification, gas separation, food safety, and biomedicine [153]. However, integrating these materials requires more complex sustainability and safety evaluations, as the composite’s fate depends on the synergistic degradation of both 2D nanosheets and the porous MOF. While ZIF-8 offers exceptional porosity and surface area, its dissolution can release zinc ions (Zn^2+^) into the environment to enhance their effectiveness against pathogens [154,155]. Consequently, the ecotoxicity and biocompatibility of MXene@ZIF-8 systems are dictated by both the metal-containing species from the MXene and the zinc dynamics of the ZIF-8 host.

Environmental applications also require careful assessment of release, persistence, transformation, and ecotoxicity. MXenes used in membranes, adsorbents, or coatings may gradually oxidize, fragment, or release metal-containing species during operation and disposal [156]. In some cases, oxidation products may be less hazardous than the parent nanosheets, but in other systems, they may alter microbial communities, aquatic organisms, or soil chemistry. Standardized testing should include realistic exposure media, chronic low-dose conditions, transformation products, and interactions with natural organic matter, salts, proteins, and microorganisms. For sustainable development, life-cycle assessment should also consider precursor mining, etchant use, washing water, solvent consumption, energy input, waste neutralization, device lifetime, and end-of-life recovery.

### 6.4. Data-Driven MXene Design and Future Outlook

Data-driven design is expected to become increasingly important in the next stage of MXene research. The large compositional space of MXenes, including carbides, nitrides, carbonitrides, ordered double-metal structures, vacancy-ordered phases, and surface-functionalized derivatives, makes experimental trial-and-error inefficient [157]. Computational screening and machine learning can help predict thermodynamic stability, electronic structure, ion adsorption, diffusion barriers, work function, mechanical properties, and catalytic activity before synthesis in nanomaterials [158]. However, predictive models require reliable datasets. At present, MXene data are fragmented because studies often report different synthesis methods, characterization metrics, testing conditions, and performance indicators. Without harmonized descriptors, machine learning may identify correlations that do not generalize across laboratories or application environments.

A stronger data infrastructure for MXenes should include both successful and unsuccessful syntheses, quantitative surface termination analysis, precursor information, processing history, stability data, and application-relevant testing conditions. Descriptors such as transition-metal identity, layer thickness, surface termination ratio, defect density, interlayer spacing, flake size, zeta potential, oxidation state, and assembly architecture should be connected to measurable outcomes and applied data-driven metrics, such as conductivity, capacitance, adsorption capacity, membrane selectivity, catalytic rate, sensing response, cytotoxicity, and long-term stability [71,159]. Such databases would support inverse design, where researchers define a desired property profile and computational tools identify promising MXene compositions and processing strategies.

Frey et al. (2019) combined elemental descriptors and high-throughput density functional theory data with positive–unlabeled learning to estimate the synthesizability of theoretically proposed MXenes and their MAX-phase precursors [160]. The model identified 18 promising MXene compounds and 20 potentially synthesizable MAX phases, demonstrating how data-driven screening can prioritize experimentally accessible candidates from a much larger theoretical space. For property prediction, Li et al. (2024) applied regression and active-learning algorithms to a database of 1757 non-centrosymmetric MXenes with nonzero band gaps [161]. Their workflow rapidly screened piezoelectric coefficients and identified candidate MXenes with strong predicted responses, followed by thermodynamic-stability evaluation of the leading structures. These examples show that machine-learning models can serve as computational surrogates for screening properties such as work function, electronic structure, ion adsorption, diffusion barriers, mechanical response, catalytic activity, and oxidation susceptibility before extensive experimental testing.

Data-driven methods can also be applied directly to experimental synthesis and processing. Wang et al. (2025) [162] combined backpropagation neural network–genetic algorithm and random forest–genetic algorithm models to optimize the preparation of tetrabutylammonium-hydroxide-modified Nb_2_CT_x_. By learning nonlinear relationships between processing conditions and measured material performance, the models reduced repeated experimental optimization and identified preparation conditions associated with improved functionality. At the material-design level, Li and Barnard (2022) [163] developed a multi-target machine-learning framework that reversed the conventional composition-to-property relationship. Instead of predicting performance from a specified MXene, the model used desired ranges of gravimetric capacity, voltage, and induced charge to identify candidate formulas, including Li_2_M_2_C and Mg_2_M_2_C, where M represented Sc, Ti, or Cr. This study provides a direct example of inverse MXene design in which application requirements are translated into candidate compositions rather than evaluated only after material selection.

The future of MXene nanomaterials will likely depend on moving from material discovery to system-level engineering. By integrating structure–property understanding with functional design, data-driven optimization, and safe-by-design principles, MXenes can progress toward practical roles in energy, environmental, electronic, biomedical, and smart material technologies.

## 7. Conclusions

MXenes have developed into one of the most versatile families of two-dimensional nanomaterials because they combine compositional diversity, metallic conductivity, hydrophilic surface chemistry, redox activity, mechanical flexibility, and solution processability. These characteristics distinguish MXenes from many other two-dimensional platforms and create broad opportunities for energy storage, catalysis, environmental remediation, sensing, electromagnetic shielding, flexible electronics, biomedical technologies, and smart functional systems. However, the performance of MXene-based materials is not determined by composition alone. It emerges from the combined effects of transition-metal identity, carbon or nitrogen sublattice, layer thickness, surface terminations, defect structure, interlayer chemistry, flake size, assembly architecture, and processing history.

This review emphasized MXenes from a structure–property and functional design perspective. Rational synthesis and processing are central because etching route, delamination method, intercalation chemistry, and storage conditions strongly influence surface termination, oxidation state, flake morphology, conductivity, and long-term stability. Structure–property relationships provide the foundation for understanding why MXenes exhibit high electronic conductivity, fast ion transport, strong interfacial activity, tunable optical and electromagnetic responses, and promising mechanical behavior. Functionalization, heterostructure construction, and composite engineering further expand the design space by enabling more precise control over charge transfer, ion accessibility, catalytic activity, adsorption selectivity, mechanical reinforcement, and environmental compatibility.

Emerging MXene technologies are increasingly moving from simple material demonstrations toward integrated systems. In energy devices, MXenes can function as active electrodes, conductive scaffolds, and ion-transport regulators. In environmental applications, they can serve as adsorbents, membranes, electrochemical separation materials, and antifouling interfaces. In sensors and flexible electronics, their conductivity and surface activity enable sensitive detection, wearable monitoring, printed devices, and electromagnetic components. Biomedical and antimicrobial applications further highlight the value of MXene photothermal behavior and surface functionalization. Across these fields, however, practical performance depends on application-specific design rather than a universal MXene formulation.

Several challenges must be addressed before MXenes can achieve broader technological translation. Oxidation, restacking, batch-to-batch variability, incomplete control of surface terminations, limited scalable manufacturing, and uncertain long-term safety remain major barriers. Future research should prioritize controlled surface chemistry, standardized characterization, reproducible synthesis, long-term stability testing, scalable processing, and safe-by-design evaluation. Data-driven approaches may also accelerate MXene development by linking synthesis conditions, structural descriptors, material properties, stability profiles, and application performance.

The next stage of MXene research should move beyond maximizing isolated properties under ideal laboratory conditions. More impactful progress will come from designing MXene systems that are stable, reproducible, processable, safe, and compatible with realistic operating environments. By integrating structure–property understanding with functional design, scalable manufacturing, and responsible development, MXene nanomaterials can progress toward reliable next-generation technologies across energy, environmental, electronic, biomedical, and multifunctional material platforms.

## Figures and Tables

**Table 1:** Representative synthesis and processing strategies for engineering MXene nanomaterials.

Strategy	Structural Outcome	Major Advantages	Limitations
HF-based selective etching	Removal of A-layer elements from MAX phases to form multilayer MXenes	Established route; effective for Ti_3_C_2_T_x_ and several carbide MXenes	Safety concerns, F-containing terminations, defect generation, oxidation risk [57]
LiF/HCl etching	In situ HF generation with intercalated Li^+^ species	Improved delamination, larger interlayer spacing, clay-like processability	Mixed terminations, residual ions, batch variability [58]
Molten-salt/Lewis acid etching	Formation of MXenes with alternative terminations such as –Cl	Expands MXene compositions; enables fluoride-free or reduced-fluoride routes	High-temperature processing, salt removal, termination control [59]
Electrochemical etching	Selective removal of A-layer under controlled electrochemical conditions	Potentially milder and more tunable than chemical etching	Limited composition range; optimization required [53]
Hydrothermal-assisted etching/intercalation	Enhanced exfoliation and improved yield	Can improve delamination and adsorption-related properties	Possible oxidation and morphology changes [37]
Intercalation-assisted delamination	Expansion of interlayer spacing using ions or organic molecules	Produces few-layer or single-layer MXene nanosheets	Sonication damage, flake-size reduction, intercalant residues [60]
Direct/CVD synthesis	Direct formation of 2D carbide or nitride phases	Access to MXene-like materials beyond MAX precursors	Early-stage development; crystallinity and scalability remain challenging [38]
Ink formulation and film assembly	Conversion of MXene dispersions into films, coatings, fibers, or printed devices	Enables scalable manufacturing and device integration	Oxidation, cracking, rheology control, substrate adhesion [61]

**Table 2:** Key structure–property relationships governing MXene functionality.

Structural Feature	Property Affected	Design Implication	Representative Relevance
Transition-metal identity	Electronic structure, redox behavior, catalytic activity	Selection of Ti, V, Nb, Mo, Ta, W, or mixed metals can tune conductivity, adsorption, and electrochemical behavior	Energy storage, catalysis, sensing [2]
Carbon/nitrogen sublattice	Bonding strength, conductivity, chemical stability	Carbides, nitrides, and carbonitrides may show different electronic and mechanical properties	Electrodes, catalysts, conductive films [92]
Layer thickness	Ion transport, mechanical stiffness, electronic behavior	M_2_X, M_3_X_2_, and M_4_X_3_ structures provide different transport and stability profiles	Batteries, membranes, mechanical films [93]
Surface terminations	Hydrophilicity, work function, ion adsorption, oxidation stability	–O, –OH, –F, and –Cl groups regulate interfacial chemistry and device performance	Sensors, membranes, electrocatalysis [94]
Interlayer spacing	Ion accessibility, swelling, molecular sieving	Intercalation and spacers can reduce restacking and improve transport	Supercapacitors, batteries, separation membranes [95]
Defects and vacancies	Active-site density, carrier scattering, reactivity	Controlled defects may enhance catalysis or adsorption, but excessive defects reduce stability	Catalysis, sensing, and environmental remediation [96]
Flake size	Conductivity, film quality, edge density, oxidation rate	Large flakes improve percolation; small flakes increase edge activity and dispersibility	Films, inks, sensors, composites [97]
Assembly architecture	Mechanical strength, conductivity, shielding, ion transport	Dense, porous, aligned, or 3D structures should be selected according to the target function	EMI shielding, electrodes, flexible devices [98]
Oxidation state and degradation products	Long-term stability and reliability	Stability must be evaluated under realistic storage and operating conditions	Films, membranes, biomedical systems [99]

**Table 3:** Representative emerging applications of MXene nanomaterials enabled by functional design.

Application Area	Representative MXene System	Functional Role	Outcomes and Advantages
Supercapacitors	Ti_3_C_2_T_x_ films and vertically aligned MXenes	Conductive redox-active electrode with fast ion transport	High volumetric capacitance and high-rate charge storage [128]
Metal-ion batteries	Ti_3_C_2_-based MXene electrodes	Ion-intercalation host and conductive scaffold	Improved Li^+^, Na^+^, K^+^, and multivalent-ion storage behavior [102]
Electrocatalysis	Mo_2_C and Ti_3_C_2_-based MXene hybrids	Electron-transfer mediator and catalytic surface	Enhanced hydrogen evolution and photocatalytic H_2_ production [129]
Water purification	Ti_3_C_2_T_x_ adsorbents and membranes	Adsorption, reduction, ion sieving, and molecular separation	Removal of dyes, Pb^2+^, Cr(VI), salts, and gases [130]
Capacitive deionization	Ti_3_C_2_T_x_ flow electrodes	Conductive ion-storage material	Ammonia removal and nutrient recovery from water streams [109]
Antibacterial membranes	Ti_3_C_2_T_x_ nanosheet membranes	Contact-mediated antibacterial and antifouling surface	Reduced microbial growth and improved membrane functionality [111]
SERS sensing	Ti_3_C_2_T_x_ MXene substrates	Charge-transfer-enhanced Raman signal amplification	Detection of small molecules and biomolecular targets [80]
Biomedical photothermal systems	Ti_3_C_2_, Nb_2_C, and MXene quantum dots	Strong NIR absorption and photothermal conversion	Tumor therapy, antimicrobial treatment, and drug-delivery platforms [131]

**Table 4:** Key translational challenges of MXene nanomaterials and engineering strategies.

Challenges	Underlying Cause	Potential Engineering Strategies	Representative Relevance
Oxidation and hydrolysis	Continuous exposure to water and dissolved oxygen; powders: residual moisture and humid storage; films: oxygen and moisture penetration through edges, cracks, and defects; composites: matrix permeability and interfacial transport pathways	Low-temperature, dark, and oxygen-restricted storage, antioxidants, pH or solvent control; powders: controlled drying and moisture-restricted storage; films: densification, lamination, barrier coatings, and encapsulation; composites: low-permeability matrices and strong interfacial bonding	Preservation of conductivity, surface chemistry, re-dispersibility, and long-term performance in inks, powders, films, electrodes, sensors, and composites [138]
Restacking of nanosheets	Strong interlayer attraction and drying-induced collapse	Intercalation, spacers, porous assembly, 3D architectures, polymer bridging	Ion transport, adsorption, membrane flux, and capacitance [144]
Batch-to-batch variability	Differences in MAX precursor, etching, washing, delamination, and storage	Standardized synthesis reporting and quality-control metrics	Reproducible conductivity, surface chemistry, and device performance [145]
Surface termination heterogeneity	Mixed –O, –OH, –F, –Cl, and residual etchant-derived groups	Controlled etching, post-treatment, molten-salt routes, termination analysis	Work function, hydrophilicity, redox activity, and interfacial behavior [146]
Limited scalable manufacturing	Laboratory-scale etching and dispersion processing	Roll-to-roll coating, printing, spray deposition, filtration, extrusion, ink formulation	Large-area films, membranes, electrodes, and coatings [147]
Safety and environmental uncertainty	Possible release, persistence, oxidation products, and biological interactions	Immobilization, exposure testing, degradation studies, life-cycle assessment	Biomedical, environmental, packaging, and water-treatment uses [148]

## Data Availability

No new data were created or analyzed in this review study. Data sharing does not apply to this article.
